# Comparative astigmatic accuracy and optical quality of SMILE, FS-LASIK, and TICL in mild-to-moderate myopia with ≥1.00 D astigmatism

**DOI:** 10.3389/fmed.2025.1692997

**Published:** 2025-12-02

**Authors:** Na Huang, Wanju Yang, Qifeng Wang, Qingsong Zhang, Yanning Yang

**Affiliations:** 1 Eye Center, Renmin Hospital of Wuhan University, Wuhan, Hubei, China; 2 Department of Refractive Surgery, Aier Eye Hospital of Wuhan University, Wuhan, China

**Keywords:** mild-to-moderate myopia, astigmatism, SMILE, FS-LASIK, TICL

## Abstract

**Purpose:**

Considering the unresolved trade-offs between astigmatic precision and optical quality in mild-to-moderate myopia with ≥1.0 D astigmatism, this study compares astigmatic correction accuracy and higher-order aberrations (HOAs) among three methods: small incision lenticule extraction (SMILE), femtosecond laser-assisted *in situ* keratomileusis (FS-LASIK), and toric implantable collamer lens (TICL) implantation.

**Methods:**

This retrospective, non-randomized comparative study enrolled 159 eyes of 159 patients. Study participants underwent either SMILE (*n* = 51 eyes), FS-LASIK (*n* = 53 eyes), or TICL implantation (*n* = 55 eyes), and their visual acuity, refractive outcomes, and optical quality parameters were assessed preoperatively and at 3 months postoperatively. Astigmatic correction efficacy was evaluated using Alpins vector analysis, with results stratified by preoperative cylinder axes.

**Results:**

At 3 months, the residual cylinder value was significantly lower in the SMILE (−0.21 ± 0.25 D) and FS-LASIK (−0.30 ± 0.23 D) groups than in the TICL group (−0.50 ± 0.26 D) (*p* < 0.05). Vector analysis demonstrated comparable target-induced astigmatism across groups. However, the TICL group exhibited significantly higher difference vectors, absolute angles of error, and index of success values than both the SMILE and FS-LASIK groups. Conversely, patients who received TICL presented lower surgically induced astigmatism, correction index, and magnitude of error values than those who received SMILE and FS-LASIK. Specifically, for against-the-rule and oblique astigmatism, the surgically induced astigmatism, magnitude of error, and correction index values were significantly higher in the SMILE and FS-LASIK groups than in the TICL group. Optical quality assessment revealed that TICL induced significantly fewer total HOAs, total coma, vertical coma, and spherical aberrations than both SMILE and FS-LASIK at 3 months.

**Conclusion:**

SMILE, FS-LASIK, and TICL implantation are all effective for correcting mild-to-moderate myopia with ≥1.0 D astigmatism. SMILE led to superior astigmatic correction accuracy compared with TICL and showed better astigmatic correction than FS-LASIK in this cohort. Meanwhile, TICL implantation induced significantly fewer HOAs than both SMILE and FS-LASIK, resulting in superior postoperative optical quality.

## Introduction

Astigmatism and myopia are highly prevalent refractive errors that can significantly affect visual quality (1). Over the last few decades, refractive surgery has been established as a safe and effective treatment for refractive errors, including astigmatism and myopia. In particular, small incision lenticule extraction (SMILE), femtosecond laser-assisted *in situ* keratomileusis (FS-LASIK), and toric implantable collamer lens (TICL) implantation have all become common choices for myopic astigmatism correction (2–5). SMILE carries the advantages of a small incision and no risk of complications related to corneal flaps; however, SMILE lacks active eye tracking. Although FS-LASIK has a relatively good eye-tracking system, a comparison of eyes with astigmatism corrected by SMILE and FS-LASIK at 7 years after the operation revealed no statistically significant difference in astigmatism (6). Finally, TICL implantation has shown good predictability in correcting astigmatism (7, 8) with a wide correction range without needing to cut the cornea. However, TICL carries the risk of postoperative lens rotation (9).

Prior studies have compared the efficacy of SMILE and TICL implantation for moderate-to-high myopia correction (10, 11). However, comparisons among the three mainstream surgical methods (SMILE, FS-LASIK, and TICL implantation) for mild-to-moderate myopia with ≥1.00 D astigmatism are lacking, and existing studies have failed to conduct comprehensive vector analyses of astigmatism and postoperative visual quality. To address this gap, in this study, we compared the postoperative visual outcomes and optical quality of patients with mild-to-moderate myopia with ≥1.00 D astigmatism after SMILE, FS-LASIK, or TICL surgery.

## Patients and methods

### Participants

For study inclusion, participants were required to have a subjective desire for spectacle independence with realistic expectations regarding surgical outcomes; an age between 18 and 45 years; documented refractive stability [defined as a ≤ 0.50 diopter (D) change in spherical equivalent (SE) over any 2-year period] for ≥2 consecutive years; a best-corrected visual acuity 0.8 on the Snellen chart; a preoperative SE ranging from −0.50 to −6.00 D; a preoperative cylinder power between −1.00 and −4.25 D; and a corneal endothelial cell density ≥2,000 cells/mm^2^. Individuals were excluded if they presented with active ocular inflammation or infection; severe dry eye syndrome (confirmed by clinical grading); uncontrolled glaucoma; or cataracts. Additional exclusion factors included ocular fundus pathologies impairing vision; severe psychiatric conditions such as uncontrolled depression or anxiety; uncontrolled systemic connective tissue diseases (e.g., rheumatoid arthritis and systemic lupus erythematosus); pregnancy or lactation; and systemic immunosuppression.

Consecutive patients undergoing refractive surgery for myopia at Aier Eye Hospital of Wuhan University between August 2024 and January 2025 were screened for eligibility. A total of 159 eyes from 159 qualified participants, all of whom satisfied the surgical inclusion criteria and granted written informed consent, were included in this investigation. Participants were assigned to one of three surgical groups as follows: (1) the SMILE group included 51 patients (51 eyes) with preoperative sphere values of −4.05 ± 1.09 D and preoperative cylinder values of −1.42 ± 0.25 D; (2) the FS-LASIK group included 53 patients (53 eyes) with preoperative sphere values of −4.17 ± 1.25 D and preoperative cylinder values of −1.41 ± 0.44 D; and (3) the TICL group included 55 patients (55 eyes) with preoperative sphere values of −4.21 ± 1.02 D and preoperative cylinder values of −1.53 ± 0.70 D. There were no statistically significant differences in the preoperative baseline parameters [age, SE, sphere, cylinder, uncorrected distance visual acuity (UDVA), and corrected distance visual acuity (CDVA)] among the three groups of patients (*p* > 0.1). Detailed preoperative demographics are presented in Table 1. This study strictly adhered to the tenets of the Declaration of Helsinki and received formal approval from the Ethics Committee of Aier Eye Hospital of Wuhan University (Approval No. 2021IRBKY1014).

**Table 1:** Baseline parameters.

Parameter	SMILE	FS-LASIK	TICL	*p*-value
Sex (female/male)	27:24	28:26	29:26	–
Age (years)	24.80 ± 6.72 (18 ~ 40)	25.11 ± 6.38 (18 ~ 43)	24.14 ± 5.98 (18 ~ 40)	0.72
Sphere, (D)	−4.05 ± 1.09	−4.17 ± 1.25	−4.21 ± 1.02	0.47
Cylinder, (D)	−1.42 ± 0.25	−1.41 ± 0.44	−1.53 ± 0.70	0.44
Spherical equivalent, D	−4.77 ± 1.13	−4.88 ± 1.25	−4.97 ± 0.99	0.64
UDVA (LogMAR)	0.97 ± 0.22	0.99 ± 0.16	1.03 ± 0.22	0.27
CDVA (LogMAR)	−0.03 ± 0.04	−0.03 ± 0.04	−0.02 ± 0.03	0.14

Data are presented as mean ± standard deviation. CDVA, corrected distance visual acuity; FS-LASIK, femtosecond laser-assisted *in situ* keratomileusis; logMAR, logarithm of the minimum angle of resolution; SMILE, small-incision lenticule extraction; TICL, toric implantable collamer lens; UDVA, uncorrected distance visual acuity.

### Surgical techniques

Eyes undergoing FS-LASIK were treated using a standardized technique. The corneal flap was generated using a SCHWIND ATOS FS200 femtosecond laser (SCHWIND Eye-Tech Solutions, Kleinostheim, Germany) with the following flap parameters: thickness, 110 μm; and diameter, 8.5 mm. Following flap creation, excimer laser ablation was performed using a SCHWIND AMARIS 500 excimer laser system (SCHWIND Eye-Tech Solutions). The optical zone diameter for the ablation procedure was set at 6.5 mm. Upon completion of laser ablation, the corneal flap was meticulously repositioned.

SMILE procedures were performed using a ZEISS VisuMax 3.0 femtosecond laser system (Carl Zeiss Meditec AG, Jena, Germany). The laser parameters included a cap thickness of 120 μm, a cap diameter of 7.5 mm, and a lenticule diameter of 6.5 mm with a 90° side-cut angle. Following laser photodisruption, the stromal lenticule was meticulously dissected using a blunt spatula and subsequently extracted intact through a superior 2.0-mm corneal incision without lenticule tear or fragmentation.

Finally, patients undergoing TICL implantation were positioned supine with the head neutrally aligned. Following standard antiseptic preparation and draping, topical anesthesia was administered. A clear corneal incision measuring 2.8 mm was made at the temporal limbus, and the TICL (STAAR Surgical AG, Nidau, Switzerland) was inserted into the anterior chamber using an injector cartridge system. An ophthalmic viscosurgical device (sodium hyaluronate 1.0%) was then instilled to maintain anterior chamber depth. Using a micro-manipulator, all four haptics were sequentially tucked beneath the iris into the posterior chamber. The lens orientation was verified against preoperative markings and adjusted to ensure perfect centration of the optic over the visual axis. Preoperatively, when the patient was seated at the slit lamp, reference markings were inserted at the 3 and 9 o’clock limbus to account for probable cyclotorsion. A Mendez gauge was utilized intraoperatively to align the TICL axis with these markings. Complete sodium hyaluronate removal was achieved through bimanual irrigation. The anterior chamber was reformed with balanced salt solution, and the corneal incision was hydrated to ensure self-sealing integrity.

All surgical groups received identical standardized topical therapy consisting of levofloxacin 0.5% eye drops administered four times daily for 2 weeks for antimicrobial prophylaxis, fluorometholone 0.1% administered four times daily during the first postoperative week for anti-inflammatory management, and preservative-free sodium hyaluronate 0.1% artificial tears applied four times daily for 4 weeks to promote ocular surface healing and lubrication.

### Routine measurements

All individuals within the study groups underwent standard postoperative management and were monitored at intervals of 1 day, 1 week, 1 month, and 3 months following the surgical intervention. Routine assessments included standard slit-lamp biomicroscopic and funduscopic examinations, CDVA and UDVA measurements, and objective and subjective refraction measurements. TICL participants underwent additional evaluations, including measurements of corneal endothelial cell density (SP-2000P; Topcon Corporation, Tokyo, Japan) and vault (Pentacam; Oculus Optikgeräte GmbH, Germany). Ocular wavefront aberrations were measured in all patients using a Pentacam system with a pupil diameter of 6 mm and then analyzed using Zernike polynomials. The Zernike coefficients of vertical coma (Z_3_^−1^), horizontal coma (Z_3_^1^), trefoil 30^°^ (Z_3_^−3^), horizontal trefoil (Z_3_^3^), and spherical aberration (SA) (Z_4_^0^) were recorded. Additionally, the root mean square (RMS) of total coma and the RMS of total higher-order aberrations, as provided by the Pentacam’s integrated software analysis, were utilized.

### Vector analysis of astigmatism

Astigmatic outcomes were evaluated using the Alpins vector analysis method (12–14). The eyes included in this study were stratified by astigmatism type based on their corneal steep meridian orientation. Eyes having with-the-rule (WTR) astigmatism were defined as having a steep corneal meridian between 60° and 120°, while those with against-the-rule and oblique astigmatism were defined as having a steep corneal meridian within 0–59° or 121–180°. Key vector parameters, including target-induced astigmatism (TIA), surgically induced astigmatism (SIA), and difference vector (DV), were derived using the ASSORT Group Analysis Calculator (available on the American Academy of Ophthalmology website at: https://www.aao.org/calculator), a tool endorsed by the International Society of Refractive Surgery. Additional parameters, namely, correction index (CI), the index of success (IOS), magnitude of error (ME), flattening effect (FE), and flattening index (FI), were calculated using the following formulas:

CI = SIA/TIA,

IOS = DV/TIA,

ME = SIA–TIA,

FE = SIA × cos(2 × AOE),

FI = FE/TIA = [SIA × cos(2 × AOE)]/TIA,

where the following are true:

- DV represents the astigmatic error remaining uncorrected after surgery, equivalent to the magnitude of the residual refractive cylinder postoperatively;
- AOE refers to the angle of error, or the angular deviation, measured in degrees, between the axis of the SIA vector and that of the TIA vector;
- FE quantifies the magnitude of the SIA vector projected onto the intended TIA meridian.

### Statistical analysis

SPSS software v. 26.0 (IBM Corp., Armonk, NY, USA) was used for all statistical analyses. The normality of continuous variable distributions was assessed using the Kolmogorov–Smirnov test. Continuous variables that either follow or closely resemble a normal distribution were expressed as mean ± standard deviation (SD). Conversely, continuous variables that do not adhere to a normal distribution were represented using the median and interquartile range (IQR) (*Q*1, *Q*3). The preoperative baseline parameters of the different surgical groups were compared using one-way analysis of variance (ANOVA). The vector analysis parameters (TIA, SIA, CI, IOS, AOE, absolute AOE [|AOE|], FI, FE, and ME), and HOAs (total HOAs, SA, and Zernike coefficients [Z_3_^−3^, Z_3_^−1^, Z_3_^1^, Z_3_^3^]) across the three groups were compared using the Kruskal–Wallis *H* test (non-parametric ANOVA). Upon the identification of substantial differences based on the Kruskal–Wallis test, subsequent pairwise comparisons were conducted using Dunn’s test with a Bonferroni adjustment. In all instances, *p* < 0.05 was considered to indicate statistical significance.

## Results

### Safety and efficacy

**Figure 1:**
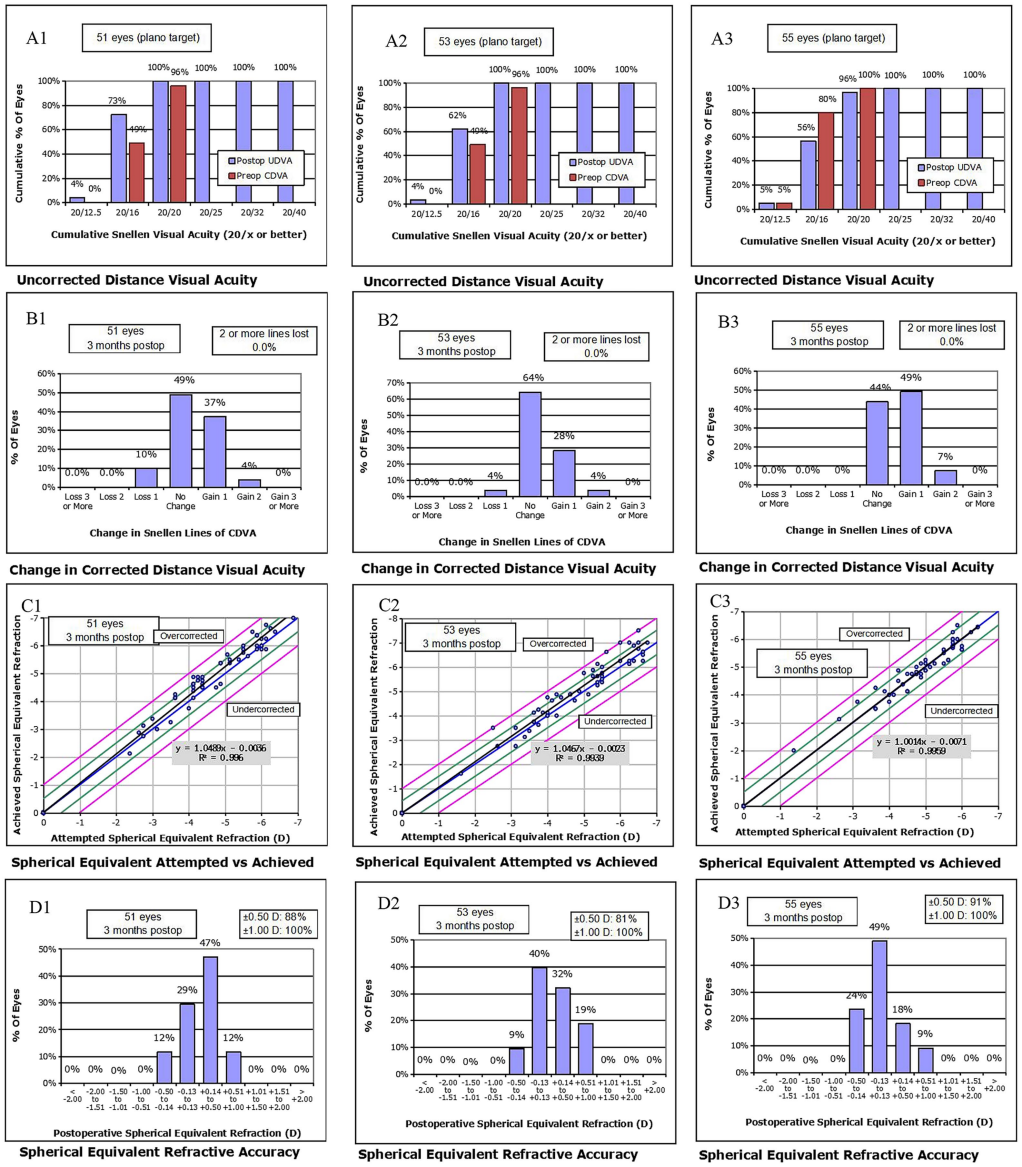
Refractive outcomes at 3 months after SMILE, FS-LASIK, and TICL implantation. Cumulative UDVA after **(A1)** SMILE, **(A2)** FS-LASIK, and **(A3**) TICL implantation. Change in CDVA after **(B1)** SMILE, **(B2)** FS-LASIK, and **(B3)** TICL implantation. Attempted vs. achieved SE refraction after **(C1)** SMILE, **(C2)** FS-LASIK, and **(C3)** TICL implantation. SE refraction after **(D1)** SMILE, **(D2)** FS-LASIK, and **(D3)** TICL implantation. SMILE, small incision lenticule extraction; FS-LASIK, femtosecond laser-assisted *in situ* keratomileusis; TICL, toric implantable collamer lens (STAAR Surgical).

All surgical procedures were completed successfully, and no complications, either intraoperative or postoperative, were noted during the final follow-up assessment. During the follow-up assessment at 3 months after surgery, the UDVA values for the SMILE, FS-LASIK, and TICL groups were −0.06 ± 0.04, −0.05 ± 0.04, and −0.04 ± 0.05 logMAR, respectively. Notably, 51 eyes (100%) in the SMILE cohort, 53 eyes (100%) in the FS-LASIK cohort, and 53 eyes (96%) in the TICL cohort achieved a UDVA of 20/20 or superior (Figure 1A). In the SMILE group, 46 eyes (90%) achieved a postoperative CDVA that was equal to or superior to the preoperative CDVA. Similarly, 55 eyes (100%) in the TICL group, and 51 eyes (96%) in the FS-LASIK group met this criterion. The postoperative CDVAs in the SMILE, FS-LASIK, and TICL groups were −0.06 ± 0.04, −0.06 ± 0.03, and −0.06 ± 0.04 logMAR, respectively, with 21 eyes (41%) in the SMILE group, 17 eyes (32%) in the FS-LASIK group, and 31 eyes (56%) in the TICL group gaining at least one line in their CDVA (Figure 1B). Comparative safety and efficacy outcomes for all three surgical groups are presented in Table 2. Although the safety index values were higher in the TICL group than in the SMILE and FS-LASIK groups, no statistically significant differences were observed in postoperative parameters (UDVA, CDVA, sphere, and efficacy index) among the groups (*p* < 0.05).

**Table 2:** Postoperative visual outcomes.

Visual outcomes	SMILE	FS-LASIK	TICL	*p*
Post-op UDVA	−0.06 ± 0.04	−0.05 ± 0.04	−0.04 ± 0.05	0.27
Post-op CDVA	−0.06 ± 0.04	−0.06 ± 0.03	−0.06 ± 0.04	0.70
Post-op sphere (D)	+0.37 ± 0.29	+0.39 ± 0.37	+0.29 ± 0.27	0.28
Post-op cylinder (D)	−0.21 ± 0.25	−0.30 ± 0.23	−0.50 ± 0.26	0.00
Safety index	1.08 ± 0.18	1.06 ± 0.13	1.11 ± 0.11	0.03
Efficacy index	1.06 ± 0.13	1.05 ± 0.15	1.06 ± 0.13	0.73

Data are presented as mean ± standard deviation. Safety index = postoperative CDVA/preoperative CDVA. Efficacy index = postoperative UDVA/preoperative CDVA. CDVA, corrected distance visual acuity; FS-LASIK, femtosecond laser-assisted *in situ* keratomileusis; logMAR, logarithm of the minimum angle of resolution; SMILE, small incision lenticule extraction; TICL, toric implantable collamer lens; and UDVA, uncorrected distance visual acuity.

### Predictability

Scatterplots of the attempted and achieved SE values, along with the corresponding regression trendlines of the three groups, are shown in Figure 1C. The proportions of eyes exhibiting postoperative SE within ±0.50 D and ±1.00 D were, respectively, 88 and 100% for the SMILE cohort, 81 and 100% for the FS-LASIK cohort, and 91 and 100% for the TICL cohort, as illustrated in Figure 1D. Furthermore, the proportions of postoperative refractive astigmatism within ±0.25, ±0.50, ±1.00, and ±1.50 D were, respectively, 69, 96, 100, and 100% for the SMILE group; 58, 92, 100, and 100% for the FS-LASIK group; and 31, 67, 91, 98, and 100% for the TICL group (Figure 1D).

Statistical analysis of refractive parameter changes over 3 months (Table 2) revealed no significant intergroup differences in manifest sphere correction (*p* = 0.28). However, statistically significant differences were observed in manifest cylinder outcomes among the groups (*p* < 0.05), indicating differential astigmatic correction efficacy between the surgical modalities.

### Vector analysis

At the 3-month postoperative assessment, the eyes in the TICL group demonstrated significantly lower SIA, CI, and ME values than those in both the SMILE and FS-LASIK groups. Conversely, the DV, |AOE|°, and IOS values were significantly higher in the TICL group than in the SMILE and FS-LASIK groups, whereas SIA was significantly lower (*p* = 0.00). Among the three groups, DV was lowest in the SMILE group, with DV showing a statistically significant improvement compared to the TICL group. Similarly, CI was significantly lower in the TICL group than in both the SMILE and FS-LASIK groups. The |AOE| values were significantly higher in the TICL group than in the SMILE and FS-LASIK groups. Among the three groups, IOS was lowest in the SMILE group, which showed a significant improvement in IOS compared to the TICL group. No statistically meaningful differences were identified between the groups for any of the other assessed parameters (Table 3).

**Table 3:** Comparison of vector analysis parameters among the SMILE, FS-LASIK, and TICL groups.

Groups	Eyes	TIA, D	SIA, D	DV, D	CI	AOE,^°^	|AOE|,^°^	IOS	FE	FI	ME, D
SMILE	51	1.12 (1.07, 1.36)	1.17 (1.02, 1.54)	0.25 (0.20, 0.42)	1.01 (0.88, 1.14)	0 (−4.78, 4.21)	4.61 (0, 6.67)	0.22 (0.16, 0.33)	0.49 (−0.59, 1.16)	0.50 (−0.50, 1.01)	0.01 (−0.13, 0.16)
FS-LASIK	53	1.14 (0.88, 1.49)	1.16 (0.89,1.54)	0.35 (0.23,0.50)	1.12 (0.86, 1.25)	0 (−4.04, 4.13)	4.13 (1.27, 7.30)	0.28 (0.19, 0.47)	0.29 (−0.69, 1.07)	0.40 (−0.62, 1.01)	0.16 (−0.16, 0.26)
TICL	55	1.14 (0.89, 1.74)	0.86 (0.62,1.30)	0.50 (0.25, 0.74)	0.74 (0.58, 0.85)	−1.29 (−8.56, 4.09)	6.08 (2.96, 12.01)	0.36 (0.22, 0.55)	0.17 (−0.38, 0.60)	0.18 (−0.31, 0.45)	−0.39 (−0.51, −0.20)
*p*		0.56	0.00	0.00	0.00	0.63	0.01	0.00	0.41	0.46	0.00

Data are expressed as *M* (*Q*1, *Q*3). |AOE|, absolute value of the angle of error; CI, correction index; D, diopters; DV, difference vector; FE, flattening effect; FI, flattening index; IOS, index of success; FS-LASIK, femtosecond laser-assisted *in situ* keratomileusis; ME, magnitude of error; SIA, surgically induced astigmatism; SMILE, small-incision lenticule extraction; TIA, target-induced astigmatism; and TICL, toric implantable collamer lens (STAAR Surgical).

Among eyes with WTR astigmatism, preoperative evaluations revealed no statistically significant variations in the SE, sphere, or cylinder values among the TICL, SMILE, and FS-LASIK groups (*p* = 0.40, *p* = 0.53, and *p* = 0.28, respectively). Similarly, no notable inter-group differences in preoperative SE, sphere, and cylinder values were observed in eyes with against-the-rule and oblique astigmatism (*p* = 0.42, *p* = 0.90, and *p* = 0.78, respectively). Postoperatively, for WTR astigmatism, SIA and ME were significantly lower in the TICL group than in both the SMILE and FS-LASIK groups. Conversely, DV, |AOE|°, and IOS were significantly higher in the TICL group than in the SMILE and FS-LASIK groups (*p* < 0.05). For the other assessed parameters, no significant differences were observed among the groups (*p* > 0.05). For against-the-rule and oblique astigmatism, SIA, CI, and ME were significantly lower in the TICL group than in the SMILE and FS-LASIK groups (*p* < 0.05). No significant differences were detected between the groups for any of the other parameters (*p* > 0.05) (Table 4).

**Table 4:** Comparative vector analysis of regular and irregular astigmatism correction in the SMILE, FS-LASIK, and TICL groups.

Groups	Eyes	TIA, D	SIA, D	DV, D	CI	AOE,^°^	|AOE|,^°^	IOS	FE	FI	ME, D
WTR											
SMILE	36	1.14 (1.09, 1.37)	1.21 (1.11, 1.52)	0.25 (0.20, 0.42)	1.01 (0.94, 1.15)	0 (−4.69, 4.43)	4.78 (0.13, 6.64)	0.22 (0.16, 0.32)	0.53 (−4.08, 1.15)	0.50 (−0.42, 1.01)	0.02 (−0.06, 0.19)
FS-LASIK	38	1.14 (0.88, 1.51)	1.27 (0.93, 1.67)	0.36 (0.24, 0.50)	1.12 (0.84, 1.25)	0 (−4.21, 4.13)	4.20 (1.42, 6.57)	0.28 (0.22, 0.46)	−0.28 (−0.71, 1.02)	−0.20 (−0.70, 0.98)	0.17 (−0.22, 0.28)
TICL	39	1.31 (1.00, 1.74)	0.88 (0.68, 1.30)	0.50 (0.25, 0.74)	0.74 (0.58, 0.84)	−2.08 (−8.56, 5.06)	6.21 (3.53, 13.62)	0.389 (0.22, 0.55)	0.24 (−0.31, 0.66)	0.19 (−0.26, 0.48)	−0.43 (−0.51, −0.22)
*p*		0.47	0.007	0.000	0.000	0.66	0.009	0.004	0.35	0.27	0.000
Non-WTR											
SMILE	15	1.07 (1.06, 1.21)	1.09 (0.88, 1.59)	0.25 (0.21, 0.41)	1.01 (0.84, 1.07)	0 (−4.34, 3.40)	4.17 (0, 6.13)	0.22 (0.18, 0.31)	−0.34 (−0.79, 1.35)	−0.31 (−0.74, 1.01)	0.01 (−0.15, 0.09)
FS-LASIK	15	1.07 (0.84, 1.30)	1.11 (0.88, 1.34)	0.25 (0.18, 0.49)	1.12 (0.98, 1.23)	0 (−2.50, 3.46)	3.00 (0, 11.30)	0.28 (0.18, 0.49)	0.82 (0.07, 1.18)	0.91 (0.15, 1.01)	0.10 (−0.02, 0.21)
TICL	16	0.89 (0.87, 1.30)	0.64 (0.60, 1.01)	0.43 (0.24, 0.61)	0.74 (0.67, 0.87)	0 (−4.53, 3.75)	3.75 (0.96, 12.70)	0.28 (0.20, 0.56)	0.45 (−0.23, 0.57)	0.24 (−0.25, 0.56)	−0.29 (−0.41, −0.08)
*p*		0.35	0.008	0.37	0.000	0.86	0.68	0.60	0.35	0.24	0.000

Data are expressed as *M* (*Q*1, *Q*3). |AOE|, absolute value of the angle of error; CI, correction index; D, diopters; DV, difference vector; FE, flattening effect; FI, flattening index; IOS, index of success; FS-LASIK, femtosecond laser-assisted *in situ* keratomileusis; ME, magnitude of error; non-WTR, against-the-rule and oblique (astigmatism); SIA, surgically induced astigmatism; SMILE, small incision lenticule extraction; TIA, target-induced astigmatism; TICL, toric implantable collamer lens; WTR, with-the-rule (astigmatism).

**Figure 2:**
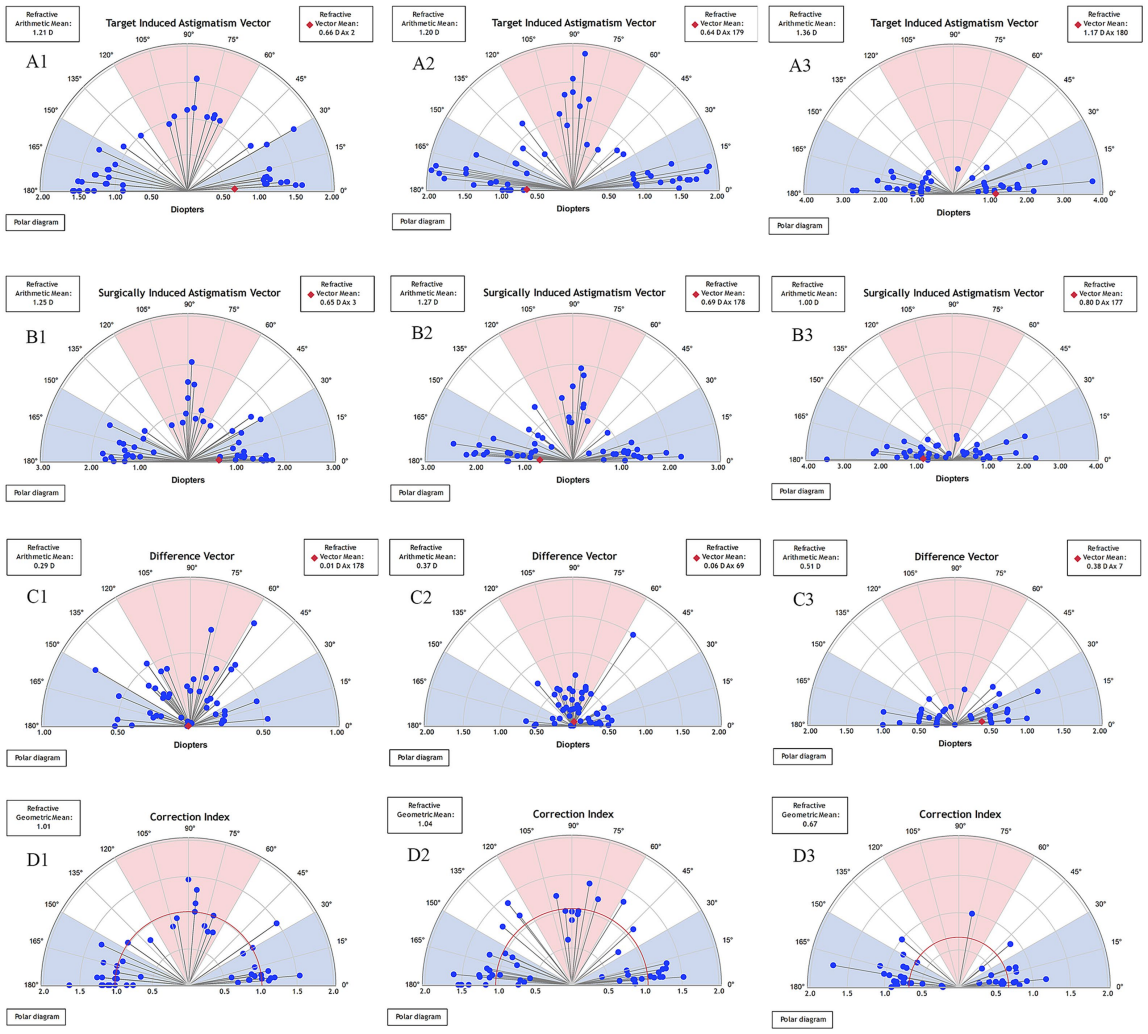
Single-angle polar plots of the target induced astigmatism vector (TIA), surgically induced astigmatism vector (SIA), difference vector (DV), and correction index (CI) at 3-month after SMILE **(A1,B1,C1,D1)**, FS-LASIK **(A2,B2,C2,D2)**, and TICL implantation **(A3,B3,C3,D3)**. The vector means are plotted as a red diamond (calculated in double-angle vector space). D, diopters; SMILE, small incision lenticule extraction; FS-LASIK, femtosecond laser-assisted *in situ* keratomileusis; TICL, toric implantable collamer lens (STAAR Surgical).

Single-angle polar plots showing the vector means of TIA, SIA, DV, and CI for SMILE, FS-LASIK, and TICL implantation during the 3-month follow-up are shown in Figure 2. The mean TIA magnitude was 1.21 D for the SMILE group, 1.20 D for the FS-LASIK group, and 1.36 D for the TICL group. The mean SIA magnitude was 1.25 D for SMILE, 1.27 D for FS-LASIK, and 1.00 D for TICL. Summated vector analysis showed that the SIA vector mean magnitude was 0.65 D at 3° for SMILE, compared to its TIA vector mean of 0.66 D at 2°. For FS-LASIK, the SIA vector mean was 0.69 D at 178° versus a TIA vector mean of 0.64 D at 179°. For TICL, the SIA vector mean was 0.80 D at 177° versus a TIA vector mean of 1.17 D at 180°. The axis alignment of the SIA and TIA vector means was within 5° for all groups. The summated DV vector mean was 0.01 D at 178° for SMILE, 0.06 D at 69° for FS-LASIK, and 0.38 D at 7° for TICL.

### HOAs

The preoperative baseline HOAs, postoperative HOAs, and changes in HOAs in the TICL, SMILE, and FS-LASIK groups are presented in Supplementary 1 and Table 5. During the 3-month follow-up period, the vertical coma and SA in the SMILE group were significantly higher than those in the FS-LASIK group and the TICL group. The RMS HOAs and coma in the TICL group were significantly lower than those in the SMILE group and the FS-LASIK group. Notably, the changes in RMS-HOAs, vertical coma, trefoil 30°, and coma were markedly lower after TICL implantation than after the SMILE and FS-LASIK procedures. The SA in the SMILE group was significantly different from that in the FS-LASIK group and the TICL group.

**Table 5:** Preoperative and postoperative changes in HOAs in eyes treated using SMILE, FS-LASIK, and TICL implantation.

Parameters	Changes in HOA of SMILE	Changes in HOA of FS-LASIK	Changes in HOA of TICL	*p*-value
RMS HOAs	0.20 (0.06, 0.31)	0.16 (0.07, 0.25)	0.01 (−0.01, 0.06)	0.00
Z_3_^3^ (μm)	−0.00 (−0.04, 0.09)	−0.03 (−0.07, 0.08)	0.07 (−0.08,0.08)	0.85
Z_3_^1^ (μm)	−0.06 (−0.13, 0.07)	−0.01 (−0.12, 0.09)	−0.00 (−0.04, 0.05)	0.39
Z_3_^−1^ (μm)	−0.26 (−0.40, −0.22)	−0.14 (−0.29, −0.06)	−0.02 (−0.08, 0.06)	0.00
Z_3_^−3^ (μm)	0.05 (−0.01, 0.12)	0.06 (−0.04, 0.15)	−0.01 (−0.03, 0.02)	0.00
SA (μm)	0.06 (0.01, 0.08)	−0.01 (−0.04, 0.05)	−0.01 (−0.03, 0.02)	0.00
Coma (μm)	0.24 (0.07, 0.37)	0.13 (0.07, 0.24)	0.03 (−0.05, 0.05)	0.00

Data are expressed as M (*Q*1, *Q*3). HOAs, higher-order aberrations; SA, spherical aberration; FS-LASIK, femtosecond laser-assisted *in situ* keratomileusis; SMILE, small incision lenticule extraction; TICL, toric implantable collamer lens (STAAR Surgical); Z_3_^−3^, trefoil 30°; Z_3_^−1^, vertical coma; Z_3_^1^, horizontal coma; Z_3_^3^, horizontal trefoil.

## Discussion

This comparative evaluation of FS-LASIK, SMILE, and TICL implantation for the correction of mild-to-moderate myopic astigmatism indicates that all three surgical modalities demonstrate favorable safety and efficacy profiles overall. However, statistically significant variations were found in their specific effects on astigmatic correction and induction of optical aberrations. This nuanced understanding of the mechanistic differences between the procedures is essential for optimizing patient selection and clinical outcomes.

Based on the visual acuity outcomes at the 3-month postoperative follow-up, high levels of UDVA and CDVA were demonstrated among all three surgical groups (FS-LASIK, SMILE, and TICL), without any statistically significant differences between groups (*p* > 0.05). This result aligns with earlier studies (15, 16) and confirms the efficacy of the three techniques in restoring visual acuity to levels comparable to, or exceeding, the preoperative best-corrected visual acuity. Notably, the proportion of eyes that achieved a gain of at least one line in CDVA was notably better in the TICL group (56%) than in the SMILE (41%) and FS-LASIK (32%) groups (*p* < 0.05). The total HOAs, vertical coma, and SA induced by TICL implantation were lower than those after corneal surgery. This reduction in aberrations is likely the primary optical explanation for the higher rate of CDVA improvement observed after TICL implantation. These observed trends align with the general observation of Aruma et al. (17), who reported that the CDVA gain was at least one line in 88% of ICL eyes versus 77% of SMILE eyes. However, our findings differ from those of Huang et al. (18) in their high-astigmatism cohort (28% in SMILE and 24% in TICL). This discrepancy likely reflects differences in study populations; the cohort of Aruma et al. (17) comprised mild-to-moderate myopia, whereas Huang et al. (18) specifically studied patients with high astigmatism. The analysis of refractive predictability revealed excellent outcomes, with a significant percentage of eyes attaining a postoperative SE within ±0.50 D: 91% in the TICL group, 88% in the SMILE group, and 81% in the FS-LASIK group, similar to the results reported by Majid et al. (19). These findings indicate favorable and comparable refractive precision among all modalities.

Regarding safety, the safety index was significantly higher in the TICL group (1.11 ± 0.11) than in the SMILE (1.08 ± 0.18) and FS-LASIK (1.06 ± 0.13) groups (*p* = 0.03). This aligns with the findings of Irene et al. (16), who reported safety indexes of 1.08 for ICL implantation and 1.03 for FS-LASIK. The superior safety index associated with TICL may be attributed to its optical principle: a posterior chamber phakic intraocular lens is used to correct refractive error close to the nodal points of the eye, potentially leading to enhanced optical quality and reduced induction of aberrations compared with corneal refractive procedures.

Astigmatic vector analysis provides a multidimensional assessment of surgical efficacy that transcends conventional SE outcomes by quantifying the alignment, magnitude, and rotational stability of astigmatism correction—parameters that directly govern postoperative visual quality (20). IOS is a validated metric for evaluating the efficacy of astigmatic correction in refractive surgery. Defined as the ratio of DV to TIA, the ideal IOS value is zero. Lower IOS values indicate a smaller proportion of residual astigmatism relative to the preoperative cylinder magnitude, signifying that the achieved astigmatic correction more closely approximates the intended outcome. In the present study, the mean IOS, DV, and |AOE|^°^ values at the 3-month follow-up were significantly higher in the TICL group than in both the SMILE and FS-LASIK groups. Meanwhile, SIA, CI, and ME were markedly lower in the TICL group than in the SMILE and FS-LASIK groups. Wan et al. (21) reported higher DV, IOS, and |AOE|^°^ values in the TICL group at the 3-month postoperative follow-up compared to the SMILE group. This suggests that the SMILE technique is superior to TICL implantation in terms of the precision in correcting both the magnitude and axis of astigmatism. In the present study, subgroup analysis further revealed that this trend was particularly pronounced among eyes with WTR astigmatism; the DV, IOS, and |AOE|^°^ values were significantly higher in the TICL group than those in the FS-LASIK and SMILE groups. In contrast, no statistically significant differences in IOS were observed among the three groups within the against-the-rule and oblique astigmatism cohort. Meanwhile, the CI values were as follows: SMILE, 1.01; FS-LASIK, 1.12; and TICL, 0.74. Our findings suggest that SMILE provides a predictable and effective approach for astigmatic correction in our cohort of patients with moderate-to-high astigmatism (≥1.00 D). This outcome should be interpreted in light of the preoperative astigmatic range, as studies involving lower levels of astigmatism, such as that by Bohac et al. (22) have reported superior astigmatic control with FS-LASIK. It is noteworthy, however, that our observation of a more pronounced induction of spherical aberration following SMILE is consistent with their report (23, 24). Regarding TICL, the early postoperative results (−0.40 D at 1 year) published by Bohac et al. (25) closely match the mean residual astigmatism in our short-term sample (−0.50 ± 0.26 D). However, their follow-up confirms the procedure’s remarkable stability by showing that these discrepancies remain non-progressive and equivalent to natural variation, so this finding of a constant residual error does not exclude a favorable long-term outcome (26). Furthermore, the elevated IOS that we observed in the WTR TICL subgroup may be attributable to postoperative lens rotation (9). Such rotation can induce misalignment between the cylindrical axis of the toric implant and the corneal astigmatic meridian, generating an undesirable cross-cylinder effect that contributes to increased residual astigmatism beyond the theoretical minimum imposed by the 0.50 D step limitation. The absence of significant IOS elevation in the against-the-rule and oblique astigmatism cohort undergoing TICL implantation may be attributed to multiple factors. First, limitations in current astigmatic vector analysis (Alpins’ method) may underestimate the TIA in eyes with irregular corneas or significant optical decentration, resulting in mathematically deflated IOS. Second, diminished statistical power in this subgroup necessitates caution in interpreting non-significance as clinical equivalence. Third, the rotational stability of TICL implantation along the horizontal axis and its compensatory effect on lenticular astigmatism neutralize its differences in IOS compared with corneal procedures. These findings suggest that, for patients with WTR astigmatism who prioritize precise correction, corneal refractive surgery should be prioritized; in contrast, for against-the-rule and oblique astigmatism patients with significant lenticular astigmatism, TICL remains a viable option.

In the present investigation, we evaluated the preoperative HOAs, postoperative HOAs, and the alterations in HOAs to discern the variations in optical performance following the three surgical procedures. Better postoperative visual quality may be associated with fewer induced aberrations (27). Nevertheless, we did not observe any notable elevation in total HOAs within the cohort that underwent TICL implantation. Similarly, Ganesh et al. (28) suggested that the absence of a significant increase in fourth-order aberrations might be related to the preservation of the corneal prolate configuration and the negative spherical aberration associated with ICL implantation. Conversely, Aruma et al. (17) reported an increase in total HOAs, predominantly characterized by vertical coma, following ICL implantation. This discrepancy may be attributed to variations in the technique employed for corneal incision and the subsequent healing process. Furthermore, we found that HOAs were elevated after both SMILE and FS-LASIK, primarily due to increases in coma and SA, in alignment with the values reported by Valencia et al. (29), Zhang et al. (30), and Huang et al. (18) at the 6-month and 1-year postoperative periods. Regarding the changes in HOAs among the three groups, TICL implantation led to a significantly lower rate of vertical coma induction compared to SMILE and FS-LASIK, with the resultant alterations in vertical coma reaching statistical significance. The limited modification of the corneal prolate surface during intraocular ICL implantation may explain this finding. This observation is consistent with the previous results reported for patients with high myopia who underwent ICL V4c implantation (31, 32).

We acknowledge several limitations of this study. First, our strict inclusion criteria limited the sample size, and the 3-month follow-up duration makes it impossible to evaluate long-term stability. Second, our evaluation of HOAs includes intrinsic methodological limitations that are crucial for the interpretation of optical quality. We did not do quantitative tear film evaluation or account for environmental factors such as room temperature and humidity, even though measurements were taken right after a blink to standardize tear film conditions. Consequently, the reported HOAs, especially following the corneal surgeries (FS-LASIK and SMILE), constitute a composite of the temporary condition of the postoperative tear film and the real corneal optics, which could have complicated our findings. Third, patient-reported outcomes—such as glare, halos, and night vision satisfaction—that are crucial for a thorough grasp of actual optical quality were absent from our study, which concentrated on objective measurements. Longer follow-up, controlled measuring settings with concurrent tear film evaluation, and the use of validated quality-of-life questionnaires would all be beneficial for future research.

## Conclusion

In summary, this comparative analysis has demonstrated that SMILE, TICL implantation, and FS-LASIK provide effective and satisfactory correction for patients with mild-to-moderate myopia with astigmatism. Importantly, SMILE demonstrated superior predictability, specifically for the astigmatic component of correction. Conversely, TICL implantation yielded the highest safety index, potentially due to its minimal induction of HOAs resulting from the preservation of the corneal structure and the optical correction occurring close to the nodal points of the eye. The distinct profiles of SMILE precision in mild-to-moderate myopic astigmatism and the optical safety advantage of TICL implantation highlight the need for an individualized approach to selecting the optimal refractive procedure. This choice should account for the corneal characteristics, astigmatic pattern, and visual demands of each patient.

## Data Availability

The original contributions presented in the study are included in the article/supplementary material, further inquiries can be directed to the corresponding author.

## Glossary

SMILE: small incision lenticule extraction
FS-LASIK: femtosecond laser-assisted *in situ* keratomileusis
TICL: toric implantable collamer lens
CDVA: corrected distance visual acuity
UDVA: uncorrected distance visual acuity
logMAR: logarithm of the minimum angle of resolution
|AOE|: absolute value of the angle of error
CI: correction index
D: diopters
DV: difference vector
FE: flattening effect
FI: flattening index
IOS: index of success
ME: magnitude of error
SIA: surgically induced astigmatism
TIA: target-induced astigmatism
WTR: with-the-rule (astigmatism)
HOAs: higher-order aberrations
SA: spherical aberration
Z_3_^−3^: trefoil 30°
Z_3_^−1^: vertical coma
Z_3_^1^: horizontal coma
Z_3_^3^: horizontal trefoil
